# Complete Draft Genome Sequence of *Escherichia coli* K802

**DOI:** 10.1128/genomeA.00934-17

**Published:** 2017-09-28

**Authors:** Gabriele R. M. Kleiner-Grote, Daniel Wibberg, Anika Winkler, Jörn Kalinowski, Karl Friehs

**Affiliations:** a Fermentation Engineering, Bielefeld University, Bielefeld, Germany; b Center for Biotechnology, Bielefeld University, Bielefeld, Germany

## Abstract

*Escherichia coli* K802 is an old strain used for cloning experiments, as well as for the production of recombinant proteins. To understand the genomic background of *E. coli* K802 better, we present here its complete draft genome sequence.

## GENOME ANNOUNCEMENT

*Escherichia coli* K802 (CGSC 5610), also designated WA802, is a K-12 derivate. Because of its impaired restriction abilities, it was often used as cloning host, especially for restriction modification (R-M) cloning experiments, as well as for the study of the bacterial R-M system against foreign DNA (1–3). Furthermore, *E. coli* K802 served as an expression strain for secretory protein production of, e.g., antibodies or single-chain fusion proteins (4, 5).

The strain was developed by multiple mutation steps based on *E. coli* K-12. Consecutive X-ray and UV light treatment, as well as phage infection steps, resulted in strain C600. This ancestor requires the supplementation of leucine, threonine, and thiamine; is unable to utilize lactose; and is resistant to coliphages T1 and T5 but sensitive toward phage *λ* (6–8). Subsequently, *met* and *gal* mutants were introduced by mating with *Hfr Calvalli* and by the use of strain 144 and P1 transduction, respectively. Finally, the nonrestricting mutant K802 was derived by spontaneous mutation (1). The strain was described as lacking McrA and McrB (9). *E. coli* K802 is available from the *E. coli* Genetic Stock Center (CGSC, New Haven, CT, USA). The currently published genotype by CGSC is as follows: [F^−^, *lacY1* or *Δ(cod-lacI)6*, *glnX44*(AS), *galK2*(Oc), *galT22*, *λ*^*−*^, *e14* mutant, *mcrA0*, *rfbC1*, *metB1*, *mcrB1*, *hsdR2*], 2016-04-20.

To establish the draft genome sequence of isolate K802, sequencing of a paired library on an Illumina MiSeq system was performed. The sequencing run (2 × 300 bp) resulted in 1,084,357 reads yielding approximately 242 Mb of sequence information. A *de novo* assembly of the obtained reads by means of the gsAssembler software version 2.8 applying default settings generated 169 contigs, accounting for a total length of 4.5 Mb featuring a GC content of 50.76%. Assembled contigs were arranged in 66 scaffolds based on the paired-end sequence information.

Gene prediction and genome annotation were performed as recently described for *E. coli* JF733 (10). Gene prediction resulted in 4,176 predicted genes, 71 tRNAs, and 2 rRNAs, which were annotated using the GenDB platform (11).

The sequence of *E. coli* K802 was analyzed and compared to that of the *E. coli* K-12 strain W3110 (GenBank accession number AP009048). Most of the published mutations could be confirmed, except for *galK2*(Oc) and *rfbC1*. Instead of *rfbC1*, a 1-bp deletion within *rfbD* was detected, resulting in a frameshift. Furthermore, the published deletion *Δ(cod-lacI)6* for K802 is different and larger than assumed. In *E. coli* K802, only the *lacY* and *lacA* genes of the *lac* operon, not *lacI* and *lacZ*, are affected by mutations. More precisely, a total region of 38.729 bp within and downstream of *lacY* is deleted in comparison to W3110, including not only the *cyn* and *cod* operon as already indicated but also the *bet* and *prp* operon, as well as several predicted and hypothetical proteins.

### Accession number(s).

The *E. coli* K802 draft genome sequence was deposited in the EMBL database under the accession numbers FMKC01000001 to FMKC01000088.
